# Long-term yogurt intake and colorectal cancer incidence subclassified by *Bifidobacterium* abundance in tumor

**DOI:** 10.1080/19490976.2025.2452237

**Published:** 2025-02-12

**Authors:** Satoko Ugai, Li Liu, Keisuke Kosumi, Hidetaka Kawamura, Tsuyoshi Hamada, Kosuke Mima, Kota Arima, Kazuo Okadome, Qian Yao, Kosuke Matsuda, Yuxue Zhong, Hiroki Mizuno, Andrew T. Chan, Wendy S. Garrett, Mingyang Song, Marios Giannakis, Edward L. Giovannucci, Xuehong Zhang, Shuji Ogino, Tomotaka Ugai

**Affiliations:** a Program in MPE Molecular Pathological Epidemiology, Department of Pathology, Brigham and Women’s Hospital, and Harvard Medical School, Boston, MA, USA; b Department of Epidemiology, Harvard T.H. Chan School of Public Health, Boston, MA, USA; c Department of Oncologic Pathology, Dana-Farber Cancer Institute, Boston, MA, USA; d Department of Surgery, Fukushima Medical University, Fukushima, Japan; e Department of Gastroenterology, Graduate School of Medicine, The University of Tokyo, Tokyo, Japan; f Clinical and Translational Epidemiology Unit, Massachusetts General Hospital and Harvard Medical School, Boston, MA, USA; g Division of Gastroenterology, Massachusetts General Hospital, Boston, MA, USA; h Department of Immunology and Infectious Diseases, Harvard T.H. Chan School of Public Health, Boston, MA, USA; i Channing Division of Network Medicine, Department of Medicine, Brigham and Women’s Hospital and Harvard Medical School, Boston, MA, USA; j Broad Institute of MIT and Harvard, Cambridge, MA, USA; k Department of Medicine, Dana-Farber Cancer Institute, Boston, MA, USA; l Department of Nutrition, Harvard T.H. Chan School of Public Health, Boston, MA, USA; m Department of Medicine, Brigham and Women’s Hospital and Harvard Medical School, Boston, MA, USA; n Department of Medical Oncology, Dana-Farber Cancer Institute and Harvard Medical School, Boston, MA, USA; o Yale University School of Nursing, Orange, CT, USA; p Cancer Immunology Program, Dana-Farber/Harvard Cancer Center, Boston, MA, USA; q Tokyo Medical and Dental University (Institute of Science Tokyo), Tokyo, Japan

**Keywords:** Nutrition, microbiome, cancer epidemiology, molecular pathological epidemiology, diet

## Abstract

Evidence suggests a tumor-suppressive effect of the intake of yogurt, which typically contains *Bifidobacterium*. We hypothesized that long-term yogurt intake might be associated with colorectal cancer incidence differentially by tumor subgroups according to the amount of tissue *Bifidobacterium*. We utilized the prospective cohort incident-tumor biobank method and resources of two prospective cohort studies. Inverse probability weighted multivariable Cox proportional hazards regression was used to assess differential associations of yogurt intake with the incidence of colorectal carcinomas subclassified by the abundance of tumor tissue *Bifidobacterium*. During follow-up of 132,056 individuals, we documented 3,079 incident colorectal cancer cases, including 1,121 with available tissue *Bifidobacterium* data. The association between long-term yogurt intake and colorectal cancer incidence differed by *Bifidobacterium* abundance (P heterogeneity = 0.0002). Multivariable-adjusted hazard ratios (HRs) (with 95% confidence intervals) in individuals who consumed ≥2 servings/week (vs. <1 serving/month) of yogurt were 0.80 (0.50–1.28) for *Bifidobacterium*-positive tumor and 1.09 (0.81–1.46) for *Bifidobacterium*-negative tumor. This differential association was also observed in a subgroup analysis of proximal colon cancer (P heterogeneity = 0.018). Long-term yogurt intake may be differentially associated with the incidence of proximal colon cancer according to *Bifidobacterium* abundance, suggesting the antitumor effect of yogurt intake on the specific tumor subgroup.

## Introduction

Yogurt, a fermented dairy food primarily containing live lactic acid bacteria, is widely considered to possess health-promoting effects.^1^ Probiotics such as yogurt are reported to be effective in the prevention of *Clostridium difficile* infection-related diarrhea^2^ and inflammatory bowel diseases.^3^ Data from long-term prospective studies suggest that yogurt intake reduces the risk of type 2 diabetes, cardiovascular disease mortality, and all-cause mortality.^4–7^ In addition, a recent meta-analysis has reported that yogurt intake is associated with a decreased risk of colorectal cancer.^8^

Evidence indicates that probiotics such as yogurt may play a cancer-preventative role via their effect on the intestinal microbiota composition and/or intestinal barrier function.^9^ Among important probiotic bacterial strains in yogurt, *Bifidobacterium* is suggested to have a tumor-suppressive effect.^10^ The biological or clinical significance of *Bifidobacterium* abundance in colorectal cancer tissue has not been fully characterized. A prior study showed that *Bifidobacterium*-positive colorectal cancer was associated with signet ring cell histology but not with other tumor characteristics or prognosis in colorectal cancer patients.^11^ The link between tissue *Bifidobacterium* abundance and the signet ring cell feature potentially implies that loss of epithelial cellular adhesion (observed in signet ring cells) might cause the entry of *Bifidobacterium* into colonic tissues. Another study also suggests that tumor *Bifidobacterium* might be an indicator of dysfunctional intestinal barriers in colorectal cancer.^12^ Additional studies are needed to characterize colorectal cancer subtypes classified by the abundance of tumor tissue *Bifidobacterium*.

Considering the important roles of both diets and the intestinal microbiota in colorectal carcinogenesis, it is of great interest to examine whether the effect of yogurt on colorectal cancer incidence differs by *Bifidobacterium* abundance. We therefore hypothesized that long-term yogurt intake might be associated with colorectal cancer incidence differentially by tumor subgroups according to the abundance of tumor tissue *Bifidobacterium*. To test this hypothesis, we utilized U.S.-wide prospective cohort studies with tumor molecular and microbial data in incident colorectal cancer cases documented in these cohort studies.

## Methods

### Study population and exposure assessments

**Figure 1.**
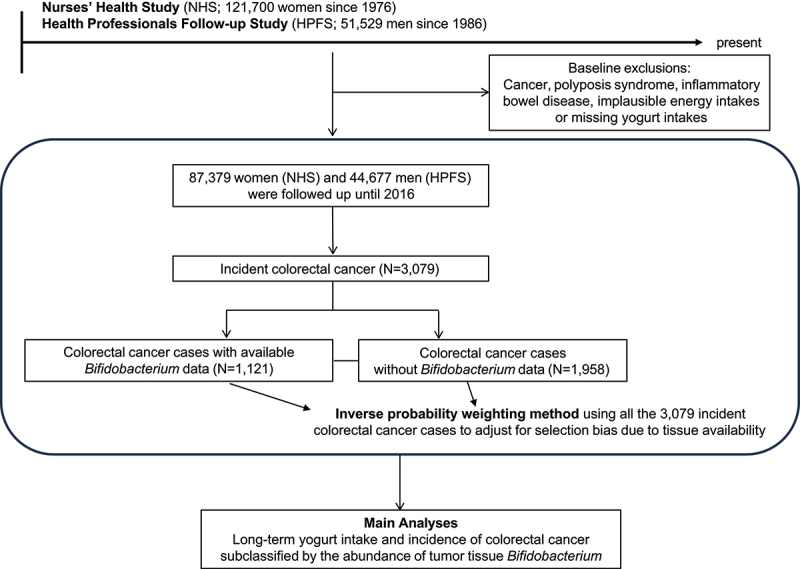
Flow diagram of the study population.

This study was based on two ongoing U.S.-wide prospective cohort studies, namely the Nurses’ Health Study (NHS) and the Health Professionals Follow-up Study (HPFS) (Figure 1), and the prospective cohort incident-tumor biobank method (PCIBM). The NHS recruited 121,700 female registered nurses aged 30 to 55 in 1976, and the HPFS enrolled 51,529 male health professionals aged 40 to 75 in 1986. Participants have been administrated questionnaires at enrollment and every two years thereafter to collect data on demographics, lifestyle factors, medical history, and disease outcomes. The follow-up rate in each cohort has been greater than 90%.

Dietary information was collected at baseline (1980 for the NHS, and 1986 for the HPFS) and nearly every 4 years thereafter using a validated semi-quantitative food frequency questionnaire (FFQ), which provided information on average daily plain and flavored yogurt intake over the preceding year. We further calculated the cumulative average intake using data from all prior cycles up to the then-latest questionnaire cycle, to best capture habitual long-term yogurt intake and reduce within-person measurement errors. A validation study showed that the mean correlation coefficient for yogurt between dietary records and FFQ was 0.97.^13^ The FFQ method had been shown to provide valid estimates of yogurt intake over the duration of the cohort studies. ^13–15^ The cumulative average yogurt intake at each FFQ cycle was calculated by averaging yogurt intake levels in all preceding (and then-current) FFQ cycles.

Participants were categorized according to the cumulative average of yogurt intake (<1 serving/month, 1–3 servings/month, 1 serving/week, and ≥ 2 servings/week). In each FFQ, we also asked about intakes of other dairy products including skim/low fat milk, whole milk, ice cream, cottage/ricotta cheese, cream cheese, other cheese, and cream. The corresponding correlation coefficients between diet records and FFQ ranged from 0.94 to 0.97 for yogurt in validation studies.^13,15^ Other dietary factors, including the intake amounts of alcohol, folate, vitamin D, processed meat, and red meat were assessed and updated using the semi-quantitative FFQs.^14,16^ In addition, lifestyle factors including adult body weight, physical activity, cigarette smoking, history of sigmoidoscopy/colonoscopy screening, family history of colorectal cancer, aspirin use, and postmenopausal hormone use (women only) were collected and updated in biennial questionnaires.^17–19^

After excluding participants with baseline cancer (except for non-melanoma skin cancer), polyposis syndrome, inflammatory bowel disease, implausible energy intake (i.e., energy intake: <600 kcal/day or >3500 kcal/day for women, <800 kcal/day or > 4200 kcal/day for men), or missing yogurt intake 87,379 women from the NHS and 44,677 men from the HPFS were included in the current analysis. The study protocol was approved by the institutional review boards of the Brigham and Women’s Hospital and Harvard T.H. Chan School of Public Health, and those of participating registries as required. The IRB number is 2019P003588.

### Ascertainment of colorectal cancer cases

Incident colorectal cancer cases were primarily identified using biennial questionnaires. Unreported colorectal cancer patient deaths were identified through the National Death Index and next of kin. We asked for written permission of patients or next of kin to obtain medical records and use tissue materials for analyses. All colorectal cancer cases were confirmed through review of medical and pathological records by a study physician who was blinded to the diet data. Information on tumor anatomic location, histologic type, and disease stage was also retrieved.

### Measurement of bifidobacterium in colorectal cancer tissue

Archival formalin-fixed paraffin-embedded (FFPE) tumor tissue blocks of confirmed colorectal cancer cases were collected from hospitals across the U.S. where the patients underwent tumor resection.^20,21^ In each case, a study pathologist (S.O.) confirmed the diagnosis of colorectal adenocarcinoma (excluding anal squamous cell carcinoma, non-epithelial tumors, and metastatic tumors to the colorectum). Genomic DNA was extracted from colorectal carcinoma tissue in whole-tissue sections of FFPE tissue blocks using QIAamp DNA FFPE Tissue Kit (Qiagen, Hilden, Germany).

To measure the amount of *Bifidobacterium* DNA in tumor tissue, we used custom TaqMan primer-probe sets for the 16S ribosomal RNA gene DNA sequence of *Bifidobacterium* at the genus level and the universal 16S rRNA sequence for the reference gene.^11,22^ The primer and probe sequences for each TaqManTM Gene Expression Assay were as follows: *Bifidobacterium* forward primer, 5′-CGGGTGAGTAATGCGTGACC-3′; *Bifidobacterium* reverse primer, 5′-TGATAGGACGCGACCCCA-3′; *Bifidobacterium* FAM probe, 5′-CTCCTGGAAACGGGTG-3′; universal 16S forward primer, 5′-CGGTGAATACGTTCCCGG-3′, universal 16S reverse primer, 5′-TACGGCTACCTTGTTACGACTT-3′; and universal 16S FAM probe, 5′-CTTGTACACACCGCCCGTC-3′. The amount of *Bifidobacterium* DNA was calculated as a relative value normalized with the universal 16S rRNA value using the 2^−ΔCt^ method (i.e., ΔC_T_ = the average C_T_ value of *Bifidobacterium* DNA − the average C_T_ value of 16S), as previously described.^11,23^ Cases with detectable *Bifidobacterium* DNA were categorized as positive, whereas cases with undetectable *Bifidobacterium* DNA were categorized as negative.

## Statistical analyses

Our primary hypothesis testing was an assessment of whether the association between yogurt consumption and colorectal cancer incidence differed by the abundance of tumor *Bifidobacterium*. All other analyses were secondary analyses. For each participant, we recorded time from the baseline to the date of colorectal cancer diagnosis, death, or end of follow-up (January 1st, 2016, for the NHS and HPFS), whichever came first. We used duplication-method time-varying Cox proportional hazards regression models for competing risk events^24^ to assess the association of yogurt intake with incidence of colorectal cancer subtypes classified by tumor *Bifidobacterium* positivity (negative vs. positive for abundant *Bifidobacterium*).

Multivariable hazard ratio (HR) was adjusted for body mass index (continuous), pack-years smoked (continuous), family history of colorectal cancer (yes vs. no), endoscopy status (yes vs. no), physical activity level (continuous), total alcohol intake (continuous), total calorie intake (continuous), total folate intake (continuous), total vitamin D intake (continuous), processed meat intake (continuous), and red meat intake (continuous), and regular aspirin use (yes vs. no). Linear trend test was calculated by continuous variables of frequency of total yogurt intake. The likelihood ratio test was used to assess statistical heterogeneity in the association of yogurt intake (continuous) with colorectal cancer incidence by tumor *Bifidobacterium* positivity (negative vs. positive).^25^ Stratified analyses by tumor anatomic subsites were also conducted. We also conducted a sensitivity analysis further adjusting for calcium intake (continuous with the ceilings of the 5th and 95th percentiles). To avoid outlier effects, we adopted the ceiling approach using the following ceiling point for each continuous covariate: 35 kg/m^2^ for body mass index; 50 pack-years for smoking; 50 metabolic equivalent task score (METS)-hours/week for physical activity; 30 g/day for alcohol; and the 5th and 95th percentile values for the intake of total calorie, total folate, total vitamin D, processed meat, and red meat.

Given that tumor microbial tissue data were not available for some colorectal cancer cases, the inverse probability weighting^26^ was used to adjust for selection bias due to tumor bacteria data availability (Figure 1). We calculated the predictive probability of *Bifidobacterium* data availability using a multivariate logistic regression model based on 3,070 incidental colorectal cancer cases in the cohorts, as previously described.^27^ We weighted each individual with *Bifidobacterium* data by multiplying the inverse of the probability. To avoid the outlier effect, we truncated the inverse of the probability at the 95th percentile. We performed a sensitivity analysis without incorporating inverse probability weighting.

All statistical analyses were performed using the SAS software (SAS Institute, Version 9.4, Cary, NC, USA). We used a two-sided alpha level of 0.005 as recommended by the expert panel of statisticians,^28^ and regarded *p* values between 0.005 and 0.05 as suggestive evidence.

## Results

### Characteristics of study participants

We utilized data from 132,056 participants (with over 3,000,000 person-years of follow-up) and 3,079 documented incidental colorectal cancer cases in the two prospective cohorts. Information on tissue *Bifidobacterium* content was available in 1,121 colorectal cancer cases within the NHS/HPFS (Figure 1). Among those, 346 cases (31%) were *Bifidobacterium*-positive, and 775 cases (69%) were *Bifidobacterium*-negative cases.

Table 1 presents age-standardized characteristics of the participants according to yogurt intake levels. Participants reporting higher yogurt intake were more likely to have higher intakes of total folate, calcium, and vitamin D, to have higher prevalence in history of sigmoidoscopy/endoscopy screening, to be more physically active, and were less likely to smoke or consume processed and red meat. The baseline characteristics of colorectal cancer cases with and without available bacteria data were generally comparable (Supplementary Table 1).

**Table 1.** Age-standardized characteristics of participants according to total yogurt intake in the Nurses’ Health Study and the Health Professionals Follow-up Study.

Characteristic^a^	Total yogurt intake (servings)			
	<1/month	1–3/month	≥4/month to < 2/week	≥2/week
Nurses’ Health Study (women)				
Age at baseline, years	59.7 (11.7)	60.8 (11.7)	64.0 (11.1)	63.0 (11.3)
Body mass index, kg/m^a^	25.0 (4.5)	25.2 (4.5)	25.1 (4.4)	24.7 (4.2)
Physical activity, METS-hours/week	10.4 (15.1)	12.3 (16.2)	14.9 (17.1)	16.6 (20.0)
Total energy intake, kcal/day	1,619 (446)	1,653 (429)	1,721 (420)	1,808 (438)
Family history of colorectal cancer, %	19	19	18	18
History of sigmoidoscopy/endoscopy, %	23	29	34	33
Regular aspirin use (2 or more tablets/week), %	41	40	40	41
Smoking, pack-years	15.9 (21.5)	11.4 (17.8)	10.4 (16.5)	9.9 (15.8)
Total folate intake, ug/day	387 (208)	432 (213)	464 (198)	493 (217)
Total calcium intake, mg/day	835 (338)	935 (344)	1024 (335)	1137 (348)
Total vitamin D intake, IU/day	324 (219)	364 (227)	389 (210)	414 (231)
Total fat intake, mg/day	64.5 (11.3)	62.1 (10.2)	59.5 (9.1)	56.2 (9.5)
Total fiber intake, g/day	16.5 (4.9)	17.7 (4.7)	18.3 (4.5)	19.1 (4.9)
Dairy intake, servings/week	12.0 (7.2)	13.4 (6.9)	15.2 (6.8)	18.3 (7.5)
Low fat dairy intake, servings/week	5.1 (5.3)	6.6 (5.3)	8.3 (5.3)	11.4 (6.2)
High fat dairy intake, servings/week	9.5 (8.1)	8.8 (7.1)	8.7 (6.8)	8.6 (6.9)
Skim milk intake, servings/week	4.3 (5.2)	5.2 (5.2)	5.8 (5.1)	6.2 (5.4)
Whole milk intake, servings/week	1.4 (3.1)	1.1 (2.6)	1.0 (2.4)	1.0 (2.5)
Ice cream intake, servings/week	1.2 (1.5)	1.1 (1.4)	1.2 (1.3)	1.2 (1.5)
Red meat intake, servings/week	2.3 (1.5)	2.0 (1.4)	1.9 (1.2)	1.8 (1.3)
Processed meat intake, servings/week	1.1 (1.4)	1.0 (1.2)	0.9 (1.1)	0.7 (1.1)
AHEI diet quality	50.2 (11.2)	53.8 (11.3)	60.0 (11.7)	58.4 (12.1)
Postmenopausal hormone use, %	40	46	50	49
Health Professionals Follow-up Study (men)				
Age at baseline, years	64.5 (11.3)	63.5 (11.3)	65.8 (11.1)	64.6 (11.4)
Body mass index, kg/m^2^	25.8 (3.4)	26.0 (3.5)	25.8 (3.6)	25.4 (3.3)
Physical activity, METS-hours/week	25.5 (23.0)	28.8 (24.4)	32.1 (25.3)	34.9 (27.8)
Total energy intake, kcal/day	1,930 (558)	1,956 (546)	2,044 (539)	2,169 (585)
Family history of colorectal cancer, %	12	12	12	12
History of sigmoidoscopy/endoscopy, %	48	53	57	53
Regular aspirin use (2 or more tablets/week), %	37	38	42	39
Smoking, pack-years	14.7 (20.0)	10.6 (16.4)	9.2 (15.0)	8.5 (14.4)
Total folate intake, ug/day	512 (248)	565 (254)	604 (250)	639 (267)
Total calcium intake, mg/day	873 (363)	950 (365)	1015 (348)	1147 (380)
Total vitamin D intake, IU/day	406 (263)	449 (266)	474 (253)	516 (279)
Total fat intake, mg/day	71.9 (12.7)	68.6 (12.0)	66.5 (11.3)	62.4 (11.9)
Total fiber intake, g/day	20.9 (6.4)	22.9 (6.3)	23.7 (6.1)	24.7 (6.9)
Dairy intake, servings/week	12.5 (8.7)	13.2 (8.0)	14.8 (8.0)	18.1 (9.2)
Low fat dairy intake, servings/week	6.4 (6.6)	7.7 (6.3)	9.3 (6.3)	12.6 (7.3)
High fat dairy intake, servings/week	8.6 (8.3)	7.8 (7.1)	8.0 (7.0)	8.0 (7.7)
Skim milk intake, servings/week	5.3 (6.4)	5.9 (6.1)	6.3 (6.0)	6.7 (6.4)
Whole milk intake, servings/week	0.9 (2.7)	0.6 (2.0)	0.5 (1.9)	0.5 (2.0)
Ice cream intake, servings/week	1.1 (1.5)	1.0 (1.2)	1.0 (1.2)	1.0 (1.3)
Red meat intake, servings/week	1.9 (1.5)	1.6 (1.3)	1.5 (1.2)	1.4 (1.2)
Processed meat intake, servings/week	1.2 (1.6)	1.0 (1.3)	0.9 (1.3)	0.8 (1.3)
AHEI diet quality	40.6 (10.0)	44.4 (10.0)	46.6 (10.0)	49.0 (10.4)

Abbreviations: AHEI, Alternate Healthy Eating Index; METS, metabolic equivalent task score.

^a^All variables are age-standardized except for age at baseline. Continuous variables are shown as mean (standard deviation). Percentage (%) indicates the proportion of participants with a specific characteristic according to total yogurt intake.

### Yogurt intake and colorectal cancer incidence by the abundance of tumor Bifidobacterium

Table 2 shows long-term yogurt intake levels and colorectal cancer incidence, overall and by the abundance of *Bifidobacterium* in tumor tissue. After adjusting potential confounding, we did not observe a significant association of yogurt intake with overall colorectal cancer incidence (P trend = 0.57; Table 2). This finding did not substantially differ by tissue *Bifidobacterium* data availability (Supplementary Table 2).

**Table 2.** Yogurt intake and colorectal cancer incidence, overall and by the abundance of tumor tissue *bifidobacterium.*

		Total yogurt intake (servings)					*P*_trend_^c^	*P*_heterogeneity_^d^
		<1/month	1–3/month	≥4/month to <2/week	≥2/week	Per serving/day		
	Person-years	1,666,863	801,420	763,055	472,977			
All colorectal cancer cases	Cases (*N* = 3,079)	1521	620	613	325			
	Age-adjusted HR (95% CI)^a^	1 (referent)	0.90 (0.82–0.99)	0.89 (0.81–0.98)	0.80 (0.70–0.90)	0.71 (0.57–0.88)	0.0017	
	Multivariable HR (95% CI)^b^	1 (referent)	0.96 (0.87–1.06)	0.98 (0.88–1.09)	0.93 (0.82–1.07)	0.94 (0.75–1.17)	0.57	
Tumor *Bifidobacterium*								
Negative	Cases (*N* = 775)	377	152	169	77			0.0002
	Age-adjusted HR (95% CI)^a^	1 (referent)	0.96 (0.78–1.19)	1.14 (0.92–1.41)	0.91 (0.70–1.22)	0.97 (0.64–1.45)	0.88	
	Multivariable HR (95% CI)^b^	1 (referent)	0.97 (0.78–1.22)	1.18 (0.95–1.48)	1.09 (0.81–1.46)	1.26 (0.87–1.82)	0.23	
Positive	Cases (*N* = 346)	186	72	60	28			
	Age-adjusted HR (95% CI)^a^	1 (referent)	0.95 (0.69–1.31)	0.88 (0.64–1.22)	0.70 (0.44–1.09)	0.45 (0.21–0.96)	0.04	
	Multivariable HR (95% CI)^b^	1 (referent)	1.05 (0.76–1.46)	0.96 (0.69–1.34)	0.80 (0.50–1.28)	0.59 (0.27–1.27)	0.18	

Abbreviations: CI, confidence interval; HR, hazard ratio.

^a^Cox proportional cause-specific hazards regression weighted by inverse probabilities based on intratumor bacteria (*Bifidobacterium*) data availability for competing risks data was used to compute HRs and 95% CIs. All analyses were stratified by age (in month), year of questionnaire return and sex.

^b^Cox proportional cause-specific hazards regression weighted by inverse probabilities based on intratumor bacteria (*Bifidobacterium*) data availability for competing risks data was used to compute HRs and 95% CIs. All analyses were stratified by age (in month), year of questionnaire return and sex. Multivariable HR was further adjusted for body mass index (continuous), pack-years smoked (continuous), family history of colorectal cancer (yes vs. no), endoscopy status (yes vs. no), physical activity level (continuous), total alcohol intake (continuous), total folate intake (continuous), total vitamin D intake (continuous), processed meat intake (continuous), red meat intake (continuous), regular aspirin use (yes vs. no), total calorie intake (continuous). To avoid outlier effects, we adopted the ceiling approach using the following ceiling point for each continuous covariate: 35 kg/m2 for body mass index; 50 pack-years for smoking; 50 metabolic equivalent task score-hours/week for physical activity; 30 g/day for alcohol; and the 5th and 95th percentile values for the intake of total calorie, total folate, total vitamin D, processed meat, and red meat.

^c^Linear trend test using the continuous variable of frequency of total yogurt intake.

^d^The likelihood ratio test was used for the test of heterogeneity of the association between total yogurt intake (continuous) and colorectal cancer risk according to the amount of *Bifidobacterium* (negative vs. positive) in the multivariable model.

In our primary hypothesis testing, the association between long-term yogurt intake and colorectal cancer incidence statistically significantly differed by *Bifidobacterium* abundance (P heterogeneity = 0.0002; Table 2). Multivariable-adjusted hazard ratios (HRs) in individuals who consumed ≥ 2 servings/week (vs. <1 serving/month) of yogurt were 0.80 [95% confidence interval (CI), 0.50–1.28] for *Bifidobacterium*-positive tumor and 1.09 (95% CI, 0.81–1.46) for *Bifidobacterium*-negative tumor.

Notably, this differential association by *Bifidobacterium* abundance was also observed in an analysis using proximal colon cancer as an outcome variable although not statistically significant at the stringent alpha level of 0.005 (P heterogeneity  = 0.018; Table 3). There was a trend of the association of yogurt intake with lower incidence of *Bifidobacterium*-positive proximal colon cancer (P trend = 0.06; the multivariable HR for yogurt consumption of ≥ 2 servings/week vs. <1 serving/month, 0.53; 95% CI, 0.27–1.06). A similar differential association was not observed in analysis of distal colon cancer or rectal cancer.

**Table 3.** Total yogurt intake and colorectal cancer incidence by the abundance of tumor tissue *bifidobacterium* and primary tumor location.

Primary tumor location	Tumor *Bifidobacterium*		Total yogurt intake (servings)					*P*_trend_^c^	*P*_heterogeneity_^d^
			<1/month	1–3/month	≥4/month to <2/week	≥2/week	Per serving/day		
Proximal colon	Negative	Cases (*N* = 386)	163	82	98	35			0.018
		Age-adjusted HR (95% CI)^a^	1 (referent)	1.10 (0.82–1.47)	1.26 (0.94–1.68)	1.24 (0.86–1.80)	1.43 (0.96–2.13)	0.08	
		Multivariable HR (95% CI)^b^	1 (referent)	1.08 (0.79–1.46)	1.19 (0.88–1.63)	1.37 (0.92–2.04)	1.51 (1.02–2.24)	0.04	
	Positive	Cases (*N* = 174)	96	37	34	7			
		Age-adjusted HR (95% CI)^a^	1 (referent)	0.79 (0.51–1.22)	0.67 (0.43–1.05)	0.41 (0.21–0.81)	0.17 (0.04–0.65)	0.01	
		Multivariable HR (95% CI)^b^	1 (referent)	0.95 (0.62–1.47)	0.81 (0.52–1.27)	0.53 (0.27–1.06)	0.30 (0.09–1.06)	0.06	
Distal colon	Negative	Cases (*N* = 261)	136	49	59	13			0.65
		Age-adjusted HR (95% CI)^a^	1 (referent)	0.89 (0.62–1.28)	1.09 (0.76–1.57)	0.66 (0.39–1.14)	0.55 (0.23–1.28)	0.17	
		Multivariable HR (95% CI)^b^	1 (referent)	0.92 (0.64–1.34)	1.26 (0.86–1.84)	0.84 (0.48–1.46)	0.82 (0.36–1.86)	0.63	
	Positive	Cases (*N* = 100)	51	23	17	8			
		Age-adjusted HR (95% CI)^a^	1 (referent)	1.24 (0.69–2.23)	1.30 (0.71–2.38)	0.66 (0.39–1.14)	0.63 (0.19–2.13)	0.46	
		Multivariable HR (95% CI)^b^	1 (referent)	1.12 (0.60–2.07)	1.32 (0.70–2.51)	0.68 (0.28–1.67)	0.57 (0.15–2.11)	0.40	
Rectum	Negative	Cases (*N* = 175)	88	30	49	7			0.50
		Age-adjusted HR (95% CI)^a^	1 (referent)	0.84 (0.51–1.37)	1.10 (0.70–1.72)	0.49 (0.27–0.89)	0.54 (0.19–1.53)	0.24	
		Multivariable HR (95% CI)^b^	1 (referent)	0.88 (0.52–1.49)	1.15 (0.70–1.88)	0.65 (0.34–1.22)	0.87 (0.31–2.48)	0.80	
	Positive	Cases (*N* = 87)	49	15	13	9			
		Age-adjusted HR (95% CI)^a^	1 (referent)	1.06 (0.52–2.15)	0.92 (0.46–1.86)	1.48 (0.69–3.14)	1.16 (0.46–2.92)	0.75	
		Multivariable HR (95% CI)^b^	1 (referent)	1.23 (0.60–2.52)	0.86 (0.40–1.85)	1.71 (0.78–3.75)	1.40 (0.53–3.67)	0.49	

Abbreviations: CI, confidence interval; HR, hazard ratio. ^a^Cox proportional cause-specific hazards regression weighted by inverse probabilities based on *Bifidobacterium* data availability for competing risks data was used to compute HRs and 95% CIs. All analyses were stratified by age (in month), year of questionnaire return and sex.

^b^Cox proportional cause-specific hazards regression weighted by inverse probabilities based on intratumor bacteria (*Bifidobacterium*) data availability for competing risks data was used to compute HRs and 95% CIs. All analyses were stratified by age (in month), year of questionnaire return and sex. Multivariable HR was further adjusted for body mass index (continuous), pack-years smoked (continuous), family history of colorectal cancer (yes vs. no), endoscopy status (yes vs. no), physical activity level (continuous), total alcohol intake (continuous), total folate intake (continuous), total vitamin D intake (continuous), processed meat intake (continuous), red meat intake (continuous), regular aspirin use (yes vs. no), total calorie intake (continuous). To avoid outlier effects, we adopted the ceiling approach using the following ceiling point for each continuous covariate: 35 kg/m2 for body mass index; 50 pack-years for smoking; 50 metabolic equivalent task score-hours/week for physical activity; 30 g/day for alcohol; and the 5th and 95th percentile values for the intake of total calorie, total folate, total vitamin D, processed meat, and red meat.

^c^Linear trend test using the continuous variable of frequency of total yogurt intake.

^d^The likelihood ratio test was used for the test of heterogeneity of the association between total yogurt intake (continuous) and cancer risk according to the amount of *Bifidobacterium* (negative vs. positive) in the multivariable model.

We conducted a sensitivity analysis further adjusting for calcium intake, which showed a similar finding to our main finding (Supplementary Table 3). A sensitivity analysis without inverse probability weighting revealed similar differential associations of yogurt intake with colorectal cancer incidence by the abundance of *Bifidobacterium* (Supplementary Table 4). In addition, to assess to what extent each covariate affects the association between yogurt intake and CRC incidence, we conducted additional sensitivity analyses excluding each covariate one by one (Supplementary Table 5). In each analysis, we observed similar findings to the main results.

## Discussion

In the two large prospective cohort studies, we tested the hypothesis that the association of long-term yogurt intake with colorectal cancer incidence might differ by the abundance of tumor tissue *Bifidobacterium*. We observed such a differential association, especially for proximal colon cancer, with a trend of the association of yogurt intake with lower incidence of *Bifidobacterium*-positive proximal colon cancer (but not *Bifidobacterium*-negative subtype). Given considerable heterogeneity in colorectal cancer by tumor subtypes,^29^ our findings suggest a potential differential influence of yogurt intake on colorectal cancer risk according to the abundance of tumor tissue *Bifidobacterium*.

It has long been believed that yogurt and other fermented milk products are beneficial for gastrointestinal health through modulating the immune system and inflammation.^30^ Most epidemiologic studies reported an inverse association of yogurt intake with colorectal cancer risk.^8,31–37^ In addition, our new findings suggest that this protective effect may be specific for *Bifidobacterium*-positive tumors.

Evidence indicates that the anti-tumor potential of yogurt may be attributed to its role in maintaining a balanced intestinal microflora, which may contribute to smooth transit of intestinal contents, competitive exclusion of deleterious microbes, maintenance of intestinal barrier function, and productions of bioactive peptides and short-chain fatty acids (SCFA).^11,12^ SCFA-producing *Bifidobacterium* can possess cancer suppression properties via antioxidant, anti-inflammatory, and immune activation effects.^13^ Srutkova et al. reported that *Bifidobacterium* promotes epithelial barrier function by reducing pro-inflammatory cytokines such as TNF (HGNC: 11892; tumor necrosis factor, tumor necrosis factor-alpha) and interleukin 6 (IL6) in a mouse model.^31^ Hence, it is conceivable that the yogurt intake can strengthen the barrier function of the intestinal mucosa, preventing bacterial infiltration into the tumor.

Studies reported that *Bifidobacterium* was detected in 30% to 35% in colorectal cancer carcinoma tissue.^11,38^ Other studies suggested that the enrichment of *Bifidobacterium* was associated with hypoxic tumor microenvironment and loss of intestinal barrier function in advanced colorectal cancer.^12,39,40^ Moreover, a previous study showed that the amount of *Bifidobacterium* in colorectal cancer tissue was associated with increased risk of anastomotic leakage after resection for colorectal cancer.^38^ Considering these studies, *Bifidobacterium* in tumor tissue could reflect impaired intestinal barrier function. Our finding suggests that yogurt intake might have a cancer preventive effect for colorectal cancer with disrupted intestinal barrier.

Previous studies indicated that the preventive effect of yogurt intake might be restricted to proximal colon cancer.^33,41^ Consistently, we observed the possible preventive effect of yogurt on *Bifidobacterium*-positive proximal colon cancer. The proximal colon is a predominant site for the conversion of primary to secondary bile acids, which is related to changes in the intestinal microbiota. It has been suggested that *Bifidobacterium* can decompose bile acids through a bile salt hydrolase activity.^42^ Therefore, our finding may suggest that yogurt intake reduces the risk of proximal colon cancer through the modulation of the microflora, including *Bifidobacterium*. Additionally, we also observed a non-statistically significant trend toward risk increase in *Bifidobacterium*-negative proximal colon cancer by yogurt. The reason for this result is not clear, and this may be a chance finding. Hence, this requires confirmation by independent studies. All of our findings should be replicated in other studies and corroborated by experimental evidence.

The current study has limitations. First, yogurt intake assessments were based on self-reported food frequency questionnaires. Although measurement errors inevitably exist, validation studies have shown reasonable validity and reproducibility of the questionnaire-based assessment of dietary intake.^43^ Second, a measurement error also exists in the FFPE tissue-based bacterial assay. Nonetheless, we rigorously validated our assay to measure *Bifidobacterium* as described in our prior study.^11^ Third, the study populations were health professionals and mostly non-Hispanic Whites. Thus, the generalizability of the findings needs to be tested in other populations. Lastly, in this study, we assessed tumor tissue *Bifidobacterium*, which is one of the probiotic bacterial strains. However, yogurt may contain other strains, such as *Lactobacillus* species. Evidence indicates that the amount of *Bifidobacterium* species can be decreased by the presence of other species within a multi-strain probiotic mixture.^44^ Therefore, future studies that comprehensively assess probiotic bacterial strains are warranted.

Our study has important strengths. First, the prospective collection of dietary intake data at multiple time points enabled us to estimate the cumulative averages to better capture information on the long-term yogurt intake of the 132,056 individuals (compared to a one-time measurement). Second, our prospective cohort design also allowed for evaluations of diets and other lifestyle factors free from differential recall bias between participants with and without incident colorectal cancer cases. Third, our comprehensive assessments of diets and other lifestyle factors enabled us to assess potential confounding of multiple other cancer risk factors. Multivariable-adjusted analyses and sensitivity analyses excluding each potential confounding factor consistently showed the protective effect of yogurt on *Bifidobacterium-*positive tumors. Although there is a possibility of residual confounding, our findings indicate that yogurt intake may reduce the risk of *Bifidobacterium*-positive CRC, even after adjusting for confounding factors. Fourth, our prospective cohort design further allowed us to control for selection bias due to tissue microbial data availability using the inverse probability weighting method and all of the 3,079 documented incident colorectal cancer cases. Fifth, the prospective cohort incident-tumor biobank method (PCIBM)^45,46^ enabled us to analyze long-term yogurt intake, other lifestyle factors, colorectal cancer incidence, and tumor tissue *Bifidobacterium* abundance and provided novel insights into the potential etiological role of yogurt intake and *Bifidobacterium* in colorectal cancer.

In conclusion, we observed a differential association of long-term yogurt intake with the incidence of colorectal cancer, especially proximal colon cancer, by the abundance of tumor tissue *Bifidobacterium*. Our findings suggest that long-term yogurt intake may lower the incidence of *Bifidobacterium-*positive proximal colorectal cancer (but not *Bifidobacterium-*negative subtype). Further studies are warranted to elucidate the potential mechanisms for the effects of long-term yogurt intake on colorectal carcinogenesis.

## Supplementary Material

Yogurt intake and CRC risk by bifido_Sup Tables_20241228.docx

## Data Availability

Further information including the procedures to obtain and access data from the Nurses’ Health Studies and Health Professionals Follow-up Study is described at https://www.nurseshealthstudy.org/researchers. (contact e-mail: nhsaccess@channing.harvard.edu) and https://sites.sph.harvard.edu/hpfs/for-collaborators/.

## Abbreviations

CI: confidence interval
FFPE: formalin-fixed paraffin-embedded
FFQ: food frequency questionnaire
HPFS: Health Professionals Follow-up Study
HR: hazard ratio
METS: metabolic equivalent task score
NHS: Nurses’ Health Study
PCIBM: prospective cohort incident-tumor biobank method
SCFA: short-chain fatty acids

## Use of standardized official symbols

We use HUGO (Human Genome Organisation) Gene Nomenclature Committee-approved official symbols (or root symbols), accompanied by unique HGNC ID where appropriate, for genes and gene products, including SLCO2A1 and TNF; all of which are described at www.genenames.org. The official gene symbols are italicized to differentiate from non-italicized gene product names and non-official colloquial names.
