# Supplementary material for: Long-term yogurt intake and colorectal cancer incidence subclassified by Bifidobacterium abundance in tumor
Source: Gut Microbes. 2025 Feb 12;17(1):2452237. doi: 10.1080/19490976.2025.2452237 (PMC11834522; doi:10.1080/19490976.2025.2452237)
Supplement: Yogurt intake and CRC risk by bifido_Sup Tables_20241228.docx [file KGMI_A_2452237_SM6580.docx]

**Supplemental Table 1**. The comparison of baseline characteristics between colorectal cancer cases with and without tumor tissue bacteria *Bifidobacterium* data

| Characteristic^*^ | Cases without *Bifidobacterium* data (N=1,958) | Cases with *Bifidobacterium* data (N=1,121) |
| --- | --- | --- |
| Age, years | 68.8 (10.2) | 68.2 (9.0) |
| Body mass index, kg/m² | 26.1 (4.3) | 25.8 (4.0) |
| Physical activity, METS-hours/week | 18.2 (20.0) | 19.3 (19.3) |
| Total energy intake, kcal/d | 1,781 (518) | 1,809 (489) |
| Family history of colorectal cancer, % | 16 | 20 |
| History of sigmoidoscopy/endoscopy, % | 35 | 36 |
| Regular aspirin use (2 or more tablets/week), % | 35 | 39 |
| Smoking, pack-years | 16.3 (21.7) | 16.6 (21.8) |
| Total folate intake, ug/d | 472 (217) | 469 (209) |
| Total calcium intake, mg/d | 939 (366) | 943 (365) |
| Total vitamin D intake, IU/d | 382 (221) | 382 (233) |
| Total fat intake, mg/d | 64.1 (12.0) | 63.9 (11.9) |
| Total fiber intake, mg/d | 19.4 (5.7) | 19.9 (6.0) |
| Dairy intake, servings/week | 13.5 (7.7) | 13.8 (7.8) |
| Low fat dairy intake, servings/week | 7.1 (5.9) | 7.3 (6.1) |
| High fat dairy intake, servings/week | 8.6 (7.7) | 8.6 (7.5) |
| Skim milk intake, servings/week | 5.2 (5.4) | 5.4 (5.5) |
| Whole milk intake, servings/week | 0.9 (2.1) | 1.0 (2.6) |
| Ice cream intake, servings/week | 1.1 (1.4) | 1.0 (1.2) |
| Red meat intake, servings/week | 1.9 (1.4) | 1.9 (1.3) |
| Processed meat intake, servings/week | 1.0 (1.4) | 1.0 (1.2) |
| AHEI diet quality | 50.3 (12.2) | 48.6 (12.0) |
| Yogurt intake, servings/week | 0.7 (1.9) | 0.5 (1.1) |

Abbreviations: AHEI, Alternate Healthy Eating Index; METS, metabolic equivalent task score.

* Continuous variables are shown as mean (standard deviation). Percentage (%) indicates the proportion of participants with a specific characteristic according to yogurt intake.

**Supplementary Table 2**. Yogurt intake and colorectal cancer incidence according to tumor *Bifidobacterium* data availability

|  |  | Total yogurt intake (servings) | | | |  | *P*_trend_^3^ |
| --- | --- | --- | --- | --- | --- | --- | --- |
|  |  | <1/month | 1-3/month | ≥4/month to  <2/week | ≥2/week | Per serving/day |  |
| All colorectal cancer cases Bifidobacterium data *Bifidobacterium* data Bifidobacterium | Cases (N=3,079) | 1521 | 620 | 613 | 325 |  |  |
|  | Age-adjusted HR (95% CI) | 1 (referent) | 0.90 (0.82-0.99) | 0.89 (0.81-0.98) | 0.80 (0.70-0.90) | 0.71 (0.57-0.88) | 0.0017 |
|  | Multivariable HR (95% CI) | 1 (referent) | 0.96 (0.87-1.06) | 0.98 (0.88-1.09) | 0.93 (0.82-1.07) | 0.94 (0.75-1.17) | 0.57 |
| *Bifidobacterium* data available cases |  |  |  |  |  |  |  |
|  | Cases (N=1,121) | 563 | 224 | 229 | 105 |  |  |
|  | Age-adjusted HR (95% CI)^1^ | 1 (referent) | 0.90 (0.77-1.05) | 0.98 (0.83-1.14) | 0.76 (0.62-0.94) | 0.71 (0.49-1.01) | 0.058 |
|  | Multivariable HR (95% CI)^2^ | 1 (referent) | 0.95 (0.81-1.12) | 1.03 (0.87-1.23) | 0.89 (0.71-1.11) | 0.90 (0.61-1.31) | 0.57 |
| *Bifidobacterium* data unavailable cases |  |  |  |  |  |  |  |
|  | Cases (N=1,958) | 958 | 396 | 384 | 220 |  |  |
|  | Age-adjusted HR (95% CI)^1^ | 1 (referent) | 0.90 (0.80-1.01) | 0.85 (0.75-0.96) | 0.81 (0.70-0.96) | 0.71 (0.55-0.93) | 0.0125 |
|  | Multivariable HR (95% CI)^2^ | 1 (referent) | 0.97 (0.85-1.10) | 0.95 (0.83-1.08) | 0.96 (0.81-1.13) | 0.96 (0.73-1.27) | 0.78 |

Abbreviations: CI, confidence interval; HR, hazard ratio.
^1^ Cox proportional cause-specific hazards regression was used to compute HRs and 95% CIs. All analyses were stratified by age (in month), year of questionnaire return, and sex.
^2^ Cox proportional cause-specific hazards regression weighted by inverse probabilities based on intratumor bacteria (*Bifidobacterium*) data availability for competing risks data was used to compute HRs and 95% CIs. All analyses were stratified by age (in month), year of questionnaire return and sex. Multivariable HR was further adjusted for body mass index (continuous), pack-years smoked (continuous), family history of colorectal cancer (yes vs. no), endoscopy status (yes vs. no), physical activity level (continuous), total alcohol intake (continuous), total folate intake (continuous), total vitamin D intake (continuous), processed meat intake (continuous), red meat intake (continuous), regular aspirin use (yes vs. no), total calorie intake (continuous). To avoid outlier effects, we adopted the ceiling approach using the following ceiling point for each continuous covariate: 35 kg/m2 for body mass index; 50 pack-years for smoking; 50 metabolic equivalent task score-hours/week for physical activity; 30 g/day for alcohol; and the 5th and 95th percentile values for the intake of total calorie, total folate, total vitamin D, processed meat, and red meat.
^3^ Linear trend test using the continuous variable of frequency of total yogurt intake.

**Supplementary Table 3**. Yogurt intake and colorectal cancer incidence by the abundance of tumor tissue *Bifidobacterium* after adjustment for calcium intake

|  |  | Total yogurt intake (servings) | | | |  | *P*_trend_^3^ | *P*_heterogeneity_^4^ |
| --- | --- | --- | --- | --- | --- | --- | --- | --- |
|  |  | <1/month | 1-3/month | ≥4/month to  <2/week | ≥2/week | Per serving/day |  |  |
|  | Person-years | 1,666,863 | 801,420 | 763,055 | 472,977 |  |  |  |
| Tumor *Bifidobacterium* |  |  |  |  |  |  |  |  |
| Negative | Cases (N=775) | 377 | 152 | 169 | 77 |  |  | 0.0002 |
|  | Age-adjusted HR (95% CI)^1^ | 1 (referent) | 0.96 (0.78-1.19) | 1.14 (0.93-1.41) | 0.92 (0.70-1.22) | 0.97 (0.64-1.45) | 0.88 |  |
|  | Multivariable HR (95% CI)^2^ | 1 (referent) | 0.97 (0.78-1.22) | 1.20 (0.96-1.50) | 1.12 (0.83-1.50) | 1.26 (0.87-1.82) | 0.23 |  |
| Positive | Cases (N=346) | 186 | 72 | 60 | 28 |  |  |  |
|  | Age-adjusted HR (95% CI)^1^ | 1 (referent) | 0.95 (0.69-1.31) | 0.88 (0.64-1.22) | 0.70 (0.44-1.09) | 0.45 (0.21-0.96) | 0.04 |  |
|  | Multivariable HR (95% CI)^2^ | 1 (referent) | 1.06 (0.77-1.47) | 0.97 (0.69-1.36) | 0.81 (0.51-1.31) | 0.59 (0.28-1.27) | 0.18 |  |

Abbreviations: CI, confidence interval; HR, hazard ratio.

^1^ Cox proportional cause-specific hazards regression weighted by inverse probabilities based on intratumor bacteria (*Bifidobacterium*) data availability for competing risks data was used to compute HRs and 95% CIs. All analyses were stratified by age (in month), year of questionnaire return and sex.
^2^ Cox proportional cause-specific hazards regression weighted by inverse probabilities based on intratumor bacteria (*Bifidobacterium*) data availability for competing risks data was used to compute HRs and 95% CIs. All analyses were stratified by age (in month), year of questionnaire return and sex. Multivariable HR was further adjusted for body mass index (continuous), pack-years smoked (continuous), family history of colorectal cancer (yes vs. no), endoscopy status (yes vs. no), physical activity level (continuous), total alcohol intake (continuous), total folate intake (continuous), total vitamin D intake (continuous), processed meat intake (continuous), red meat intake (continuous), regular aspirin use (yes vs. no), total calorie intake (continuous), and calcium intake (continuous). To avoid outlier effects, we adopted the ceiling approach using the following ceiling point for each continuous covariate: 35 kg/m2 for body mass index; 50 pack-years for smoking; 50 metabolic equivalent task score-hours/week for physical activity; 30 g/day for alcohol; and the 5th and 95th percentile values for the intake of total calorie, total folate, total vitamin D, processed meat, red meat, and calcium.
^3^ Linear trend test using the continuous variable of frequency of total yogurt intake.
^4^ The likelihood ratio test was used for the test of heterogeneity of the association between total yogurt intake (continuous) and colorectal cancer risk according to the amount of *Bifidobacterium* (negative vs. positive) in the multivariable model.

**Supplementary Table 4**. Yogurt intake and colorectal cancer incidence by the abundance of tumor tissue *Bifidobacterium* without inverse probability weighting

|  | |  |  | Total yogurt intake (servings) | | | | *P*_trend_^3^ | *P*_heterogeneity_^4^ |
| --- | --- | --- | --- | --- | --- | --- | --- | --- | --- |
|  |  |  |  | < 1/month | 1-3/month | 1/week | ≥ 2/week |  |  |
| Tumor *Bifidobacterium* | Negative | | Cases (N=775) | 377 | 152 | 169 | 77 |  | 0.03 |
|  |  | | Age-adjusted HR (95% CI)^1^ | 1 (referent) | 0.93 (0.77-1.13) | 1.09 (0.91-1.32) | 0.85 (0.66-1.09) | 0.48 |  |
|  |  | | Multivariable HR (95% CI)^2^ | 1 (referent) | 0.97 (0.79-1.19) | 1.15 (0.94-1.41) | 1.01 (0.77-1.31) | 0.68 |  |
|  | Positive | | Cases (N=346) | 186 | 72 | 60 | 28 |  |  |
|  |  | | Age-adjusted HR (95% CI)^1^ | 1 (referent) | 0.86 (0.65-1.13) | 0.69 (0.51-0.94) | 0.60 (0.40-0.89) | 0.004 |  |
|  |  | | Multivariable HR (95% CI)^2^ | 1 (referent) | 0.96 (0.72-1.27) | 0.74 (0.54-1.02) | 0.69 (0.45-1.04) | 0.04 |  |

Abbreviations: CI, confidence interval; HR, hazard ratio.

^1^ Cox proportional cause-specific hazards regression weighted by inverse probabilities based on intratumor bacteria (*Bifidobacterium*) data availability for competing risks data was used to compute HRs and 95% CIs. All analyses were stratified by age (in month), year of questionnaire return and sex.
^2^ Cox proportional cause-specific hazards regression weighted by inverse probabilities based on intratumor bacteria (*Bifidobacterium*) data availability for competing risks data was used to compute HRs and 95% CIs. All analyses were stratified by age (in month), year of questionnaire return and sex. Multivariable HR was further adjusted for body mass index (continuous), pack-years smoked (continuous), family history of colorectal cancer (yes vs. no), endoscopy status (yes vs. no), physical activity level (continuous), total alcohol intake (continuous), total folate intake (continuous), total vitamin D intake (continuous), processed meat intake (continuous), red meat intake (continuous), regular aspirin use (yes vs. no), total calorie intake (continuous). To avoid outlier effects, we adopted the ceiling approach using the following ceiling point for each continuous covariate: 35 kg/m2 for body mass index; 50 pack-years for smoking; 50 metabolic equivalent task score-hours/week for physical activity; 30 g/day for alcohol; and the 5th and 95th percentile values for the intake of total calorie, total folate, total vitamin D, processed meat, and red meat.
^3^ Linear trend test using the continuous variable of frequency of total yogurt intake.
^4^ The likelihood ratio test was used for the test of heterogeneity of the association between total yogurt intake (continuous) and colorectal cancer risk according to the amount of *Bifidobacterium* (negative vs. positive) in the multivariable model.

**Supplementary Table 5**. Yogurt intake and colorectal cancer incidence by the abundance of tumor tissue *Bifidobacterium* after excluding each covariate.

| Analysis with an excluded variable | Tumor *Bifidobacterium* | Total yogurt intake (servings) with corresponding multivariable HR (95% CI)^1^ | | | | | *P*_trend_^2^ | *P*_heterogeneity_^3^ |
| --- | --- | --- | --- | --- | --- | --- | --- | --- |
|  |  | <1/month | 1-3/month | ≥4/month to <2/week | ≥2/week | Per serving/day |  |  |
| Physical activity |  |  |  |  |  |  |  | 0.0002 |
|  | Negative | 1 (referent) | 0.97 (0.78-1.21) | 1.18 (0.94-1.47) | 1.08 (0.80-1.45) | 1.20 (0.82-1.76) | 0.35 |  |
|  | Positive | 1 (referent) | 1.05 (0.76-1.45) | 0.95 (0.68-1.33) | 0.79 (0.49-1.26) | 0.56 (0.26-1.20) | 0.13 |  |
| History of sigmoidoscopy/  endoscopy |  |  |  |  |  |  |  | 0.0002 |
|  | Negative | 1 (referent) | 0.96 (0.77-1.20) | 1.15 (0.92-1.44) | 1.07 (0.79-1.43) | 1.19 (0.80-1.76) | 0.40 |  |
|  | Positive | 1 (referent) | 1.04 (0.75-1.44) | 0.94 (0.67-1.31) | 0.78 (0.49-1.26) | 0.55 (0.25-1.19) | 0.13 |  |
| Pack-years smoke |  |  |  |  |  |  |  | 0.0002 |
|  | Negative | 1 (referent) | 0.96 (0.77-1.20) | 1.17 (0.93-1.46) | 1.08 (0.80-1.44) | 1.20 (0.82-1.76) | 0.35 |  |
|  | Positive | 1 (referent) | 1.04 (0.75-1.44) | 0.94 (0.68-1.32) | 0.78 (0.49-1.26) | 0.55 (0.26-1.20) | 0.13 |  |
| Total folate intake |  |  |  |  |  |  |  | 0.0002 |
|  | Negative | 1 (referent) | 0.97 (0.78-1.22) | 1.19 (0.95-1.48) | 1.09 (0.82-1.47) | 1.22 (0.84-1.78) | 0.30 |  |
|  | Positive | 1 (referent) | 1.05 (0.76-1.46) | 0.96 (0.69-1.34) | 0.80 (0.50-1.28) | 0.57 (0.27-1.23) | 0.15 |  |
| Total vitamin D intake |  |  |  |  |  |  |  | 0.0002 |
|  | Negative | 1 (referent) | 0.97 (0.78-1.21) | 1.17 (0.94-1.47) | 1.08 (0.81-1.45) | 1.21 (0.83-1.77) | 0.33 |  |
|  | Positive | 1 (referent) | 1.05 (0.76-1.45) | 0.95 (0.68-1.33) | 0.79 (0.49-1.27) | 0.56 (0.26-1.21) | 0.14 |  |
| Red meat intake |  |  |  |  |  |  |  | 0.0002 |
|  | Negative | 1 (referent) | 0.97 (0.78-1.20) | 1.17 (0.94-1.46) | 1.07 (0.80-1.43) | 1.19 (0.81-1.73) | 0.38 |  |
|  | Positive | 1 (referent) | 1.04 (0.76-1.44) | 0.94 (0.68-1.32) | 0.78 (0.49-1.25) | 0.55 (0.25-1.18) | 0.12 |  |
| Processed meat intake |  |  |  |  |  |  |  | 0.0002 |
|  | Negative | 1 (referent) | 0.97 (0.78-1.21) | 1.18 (0.94-1.47) | 1.08 (0.81-1.45) | 1.21 (0.82-1.76) | 0.34 |  |
|  | Positive | 1 (referent) | 1.05 (0.76-1.45) | 0.95 (0.68-1.33) | 0.79 (0.49-1.27) | 0.56 (0.26-1.21) | 0.14 |  |

Abbreviations: CI, confidence interval; HR, hazard ratio.
^1^ Cox proportional cause-specific hazards regression weighted by inverse probabilities based on intratumor bacteria (*Bifidobacterium*) data availability for competing risks data was used to compute HRs and 95% CIs. All analyses were stratified by age (in month), year of questionnaire return and sex. Multivariable HR was further adjusted for body mass index (continuous), pack-years smoked (continuous), family history of colorectal cancer (yes vs. no), endoscopy status (yes vs. no), physical activity level (continuous), total alcohol intake (continuous), total folate intake (continuous), total vitamin D intake (continuous), processed meat intake (continuous), red meat intake (continuous), regular aspirin use (yes vs. no), total calorie intake (continuous) except for the excluded covariate. To avoid outlier effects, we adopted the ceiling approach using the following ceiling point for each continuous covariate: 35 kg/m2 for body mass index; 50 pack-years for smoking; 50 metabolic equivalent task score-hours/week for physical activity; 30 g/day for alcohol; and the 5th and 95th percentile values for the intake of total calorie, total folate, total vitamin D, processed meat, and red meat.

^2^ Linear trend test using the continuous variable of frequency of total yogurt intake.
^3^ The likelihood ratio test was used for the test of heterogeneity of the association between total yogurt intake (continuous) and colorectal cancer risk according to the amount of *Bifidobacterium* (negative vs. positive) in the multivariable model.
